# Vancomycin-Stabilized Platinum Nanoparticles with Oxidase-like Activity for Sensitive Dopamine Detection

**DOI:** 10.3390/biom13091312

**Published:** 2023-08-26

**Authors:** Yuzhen Xue, Kai Liu, Mingyue Gao, Tiantian Zhang, Longgang Wang, Yanshuai Cui, Xianbing Ji, Guanglong Ma, Jie Hu

**Affiliations:** 1 State Key Laboratory of Metastable Materials Science and Technology, Hebei Key Laboratory of Nano-Biotechnology, Hebei Key Laboratory of Applied Chemistry, Yanshan University, Qinhuangdao 066004, China; xueyz@ihep.ac.cn (Y.X.); d202210477@xs.ustb.edu.cn (K.L.); gmy1999@stumail.ysu.edu.cn (M.G.); zttguan@stumail.ysu.edu.cn (T.Z.); hujie@ysu.edu.cn (J.H.); 2 Department of Environmental Engineering, Hebei University of Environmental Engineering, Qinhuangdao 066102, China; cuiyanshuai@hebuee.edu.cn (Y.C.); jixianbing@hebuee.edu.cn (X.J.); 3 Centre for Cancer Immunology, Faculty of Medicine, University of Southampton, Southampton SO166YD, UK; mguanglong@gmail.com

**Keywords:** dopamine, nanozyme, platinum nanoparticles, colorimetric detection

## Abstract

The development of efficient, reliable, and sensitive dopamine detection methods has attracted much attention. In this paper, vancomycin-stabilized platinum nanoparticles (Van-Pt_n_ NPs, n = 0.5, 1, 2) were prepared by the biological template method, where n represented the molar ratio of vancomycin to Pt. The results show that Van-Pt_2_ NPs had oxidase-like activity and peroxidase-like activity, and the mechanism was due to the generation of reactive oxygen ^1^O_2_ and OH. Van-Pt_2_ NPs exhibited good temperature stability, storage stability, and salt solution stability. Furthermore, Van-Pt_2_ NPs had almost no cytotoxicity to A549 cells. More importantly, the colorimetric detection of DA in human serum samples was performed based on the oxidase-like activity of Van-Pt_2_ NPs. The linear range of DA detection was 10–700 μM, and the detection limit was 0.854 μM. This study establishes a rapid and reliable method for the detection of dopamine and extends the application of biosynthetic nanoparticles in the field of biosensing.

## 1. Introduction

Dopamine (DA) is a neurotransmitter that regulates a variety of physiological processes in the human body, such as emotion, memory, behavior, and learning [1,2]. It can also affect the body’s metabolism, such as appetite, sleep, temperature regulation, and exercise capacity. Abnormal dopamine secretion can lead to cognitive dysfunction, Parkinson’s disease, anxiety, obesity, and so on [3]. In addition, drug consumption can also lead to excessive secretion of DA, which leads to drug addiction. Thus, it is important to determine the concentration of DA. At present, the analytical methods for the detection of DA include colorimetric analysis [4], fluorescence analysis [5], electrochemical analysis [6], and photothermal analysis [7]. Compared with other analysis methods, the colorimetric method has the advantages of simple operation, low cost, and good visibility. In colorimetric analysis, it is necessary to add natural enzymes to accelerate the reaction. Natural enzymes have attracted much attention due to their high catalytic activity, high selectivity, and high specificity. However, the application of natural enzymes is restricted by shortcomings such as difficult storage and easy inactivation [8].

Compared with natural enzymes, nanozymes have the advantages of easy storage, simple preparation, and easy control. At present, a large number of nanomaterials with oxidase-like and peroxidase-like activities have been developed, including metal oxide nanomaterials [9], carbon-based nanomaterials [10], metal–organic frameworks [11] and noble metal nanomaterials [12]. Noble metal nanomaterials have a large specific surface area and unique D-layer electron orbitals, which can make reactants more inclined to adsorb on their surface and thus form reactive intermediates [13]. Therefore, noble metal nanomaterials have the advantages of high catalytic activity and enzyme-like activity. Based on their enzyme-like activities, noble metal nanozymes can be used to construct economical DA detection platforms. Platinum nanoparticles (Pt NPs) are among the most effective catalysts due to their superior enzyme-like activity, good biocompatibility, and catalytic chemiluminescence properties, which can serve as mimic enzymes for oxidase, peroxidase, catalase, and superoxide dismutase [14]. Wang et al. [15] synthesized an atomically dispersed diatomic active site nanozymes (FeN_3_/PtN_4_-single-atom nanozymes (SAzyme)) through a two-part pyrolysis process. This FeN_3_/PtN_4_-SAzyme showed good DA colorimetric detection performance. The linear detection range of DA was 1–10 μM, and the detection limit was 0.109 μM. Li et al. [16] prepared Pt/NH101-MIL-2 hybrid nanozymes with bimetallic catalytic centers by forming a coordination bond between Pt nanoparticles (Pt NPs) and -NH_2_ on a metal–organic skeleton (MOF). The catalytic activity of Pt/NH_2_-MIL-101 was increased by 1.5 times, and the detection limit of DA molecule was only 0.42 μM by colorimetry.

However, nanozymes for DA detection are mainly synthesized via physical and chemical methods, resulting in nanozymes that are generally toxic, unstable, aggregated, and environmentally unfriendly. In the face of these problems, bioregulated synthesis using biological molecules has attracted much attention due to its capacity to control the structure of nanomaterials and improve biocompatibility. As an antibiotic, vancomycin has good water solubility. In addition, its own hydroxyl group and amino group can not only form hydrogen bonds with water but also form weak forces with Pt^2+^ and Pt atoms so as to achieve the effects of a mineralizer and a stabilizer. However, the facile detection of DA by vancomycin-stabilized platinum nanoparticles has not been reported.

Herein, vancomycin-stabilized platinum nanoparticles (Van-Pt_n_ NPs n = 0.5, 1, 2) were prepared by adding different amounts of K_2_PtCl_4_ with vancomycin under the reducing agent. This was carried out to investigate the structure characterization, enzyme-like activity, catalytic kinetics, and catalytic mechanism of the synthesized Van-Pt_n_ NPs. Among them, the Van-Pt_2_ NPs showed good thermal stability with long storage time and storage stability. Based on the oxidase-like activity of Van-Pt_2_ NPs, a simple and sensitive method for DA detection was developed. In addition, Van-Pt_2_ NPs also exhibited good biocompatibility. The Van-Pt_2_ NPs in this study provides a novel strategy for DA detection with great potential application.

## 2. Materials and Methods

The materials, synthetic method for Van-Pt_n_ NPs (n = 0.5, 1, 2), material characterization, enzyme activity-testing experiments, cytocompatibility experiments, and DA assays are described in detail in the supporting information.

## 3. Results

### 3.1. Preparation and Structure Analysis

**Figure 1:**
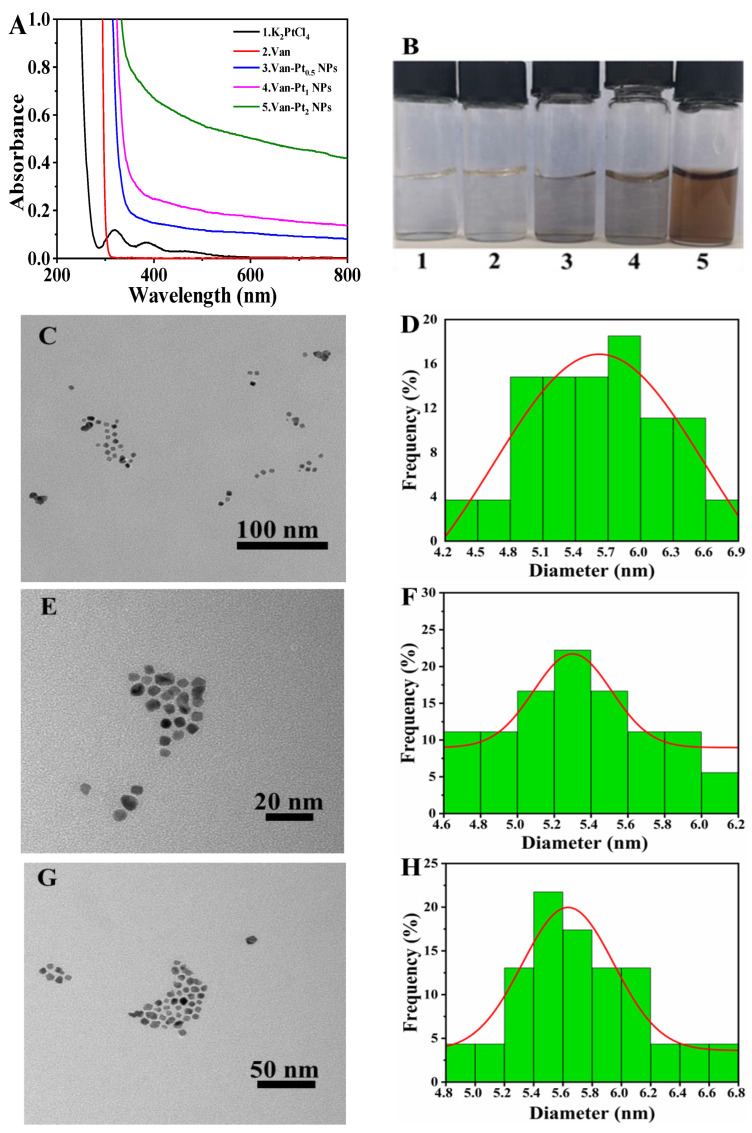
(**A**) UV–vis spectra of different substances; (**B**) the corresponding image of (**A**); TEM pictures and corresponding particle size statistics: (**C**,**D**) Van-Pt_0.5_ NPs, (**E**,**F**) Van-Pt_1_ NPs, (**G**,**H**) Van-Pt_2_ NPs.

The synthesis of Van-Pt_n_ NPs can be preliminarily characterized by the disappearance or appearance of specific peaks. As shown in Figure 1A, K_2_PtCl_4_ has two characteristic absorption peaks at 392 nm and 329 nm. After vancomycin was co-incubated with K_2_PtCl_4_ for 12 h, NaBH_4_ was added to reduce Pt^2+^ to form Pt for 12 h, and the two characteristic absorption peaks of Pt^2+^ disappeared. This is similar to the characterization of Pt NPs prepared by Li et al. [17]. In addition, Figure 1B shows that with the increase in the molar ratio of vancomycin to K_2_PtCl_4_, the absorbance of the synthesized Van-Pt_n_ NPs also increased and the color of the sample deepened. Among them, Van-Pt_2_ NPs have the highest absorbance. The morphology of Van-Pt_n_ NPs and the size of their particles were characterized by TEM [18]. The particle sizes were 5.48 ± 0.6 nm for Van-Pt_0.5_ NPs (Figure 1C,D), 5.15 ± 0.3 nm for Van-Pt_1_ NPs (Figure 1E,F), and 5.63 ± 0.4 nm for Van-Pt_2_ NPs (Figure 1G,H), respectively. Thus, there is no significant difference in particle size and morphology between Van-Pt_n_ NPs at the three ratios.

**Figure 2:**
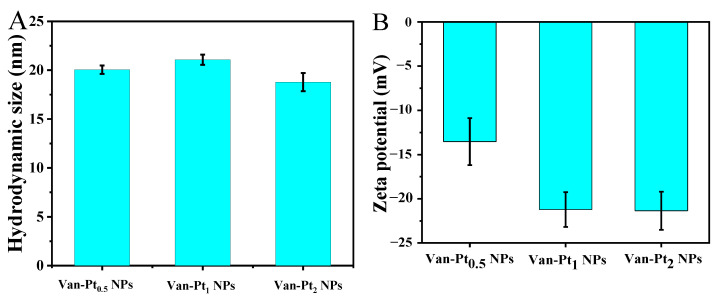
DLS characterization of Van-Pt_n_ NPs (n = 0.5, 1, 2): (**A**) hydrodynamic size; (**B**) zeta potential.

The catalysis of nanozymes is mainly carried out in aqueous solution. The states of nanomaterials are measured by DLS [19]. As depicted in Figure 2A, the hydrodynamic sizes of Van-Pt_0.5_ NPs, Van-Pt_1_ NPs, and Van-Pt_2_ NPs were 20.05 ± 0.43 nm, 21.07 ± 0.51 nm, and 18.78 ± 0.94 nm, respectively. As shown in Figure 2B, the zeta potentials of Van-Pt_0.5_ NPs, Van-Pt_1_ NPs, and Van-Pt_2_ NPs were −13.53 ± 2.66 mV, −21.22 ± 1.97 mV, and −21.35 ± 2.15 mV, respectively. Among them, Van-Pt_2_ NPs had the largest absolute zeta potential value, which was conducive to the better stability and dispersion of Van-Pt_2_ NPs in aqueous solution.

Van-Pt_2_ NPs were used as a representative to characterize the elemental composition and valence states of Van-Pt_2_ NPs by XPS [20]. Figure S1A has four elements: C, N, O, and Pt. The three elements—C, N, and O—were all derived from the biological template—vancomycin—and Pt was reduced from K_2_PtCl_4_. As shown in Figure S1B, the corresponding binding energies of Pt 4f_7/2_ and Pt 4f_5/2_ are 71.2 eV and 74.5 eV, respectively. This result was very similar to the characterization of highly dispersed Pt NPs on N-doped ordered mesoporous carbon prepared by Sheng et al. [21] Thus, the Pt^2+^ in K_2_PtCl_4_ was reduced from +2 valence to 0 valence, which proved the successful preparation of Van-Pt_2_ NPs.

Figure S1C shows that C 1s binding energies are 284.6 eV, 286.3 eV, and 288.5 eV, which correspond to C–C [22], C–O [23], and C=O [24], respectively. The binding energy of N 1s in Figure S1D is 399.5 eV, which is consistent with the binding energy of N–C in nitrogen-doped graphene nanoribbon reported by Yamada Y et al. [25]. The elements C and N are provided by vancomycin. Therefore, the successful recombination of vancomycin and Pt NPs was confirmed via XPS determination. According to the XRD patterns in Figure S1E, the diffraction angles of Van-Pt_2_ NPs are 39.87°, 46.26°, 67.68°, 81.43°, and 86.12°, which correspond to the (111), (200), (220), (311), and (222) crystal planes, respectively. Compared with the reference code of Pt 01-001-1194, it is found that Pt NPs in the synthesized Van-Pt_2_ NPs had a face-centered cubic crystal structure. In short, these results suggest the successful preparation of Van-Pt_2_ NPs.

### 3.2. Enzyme-Like Property

**Figure 3:**
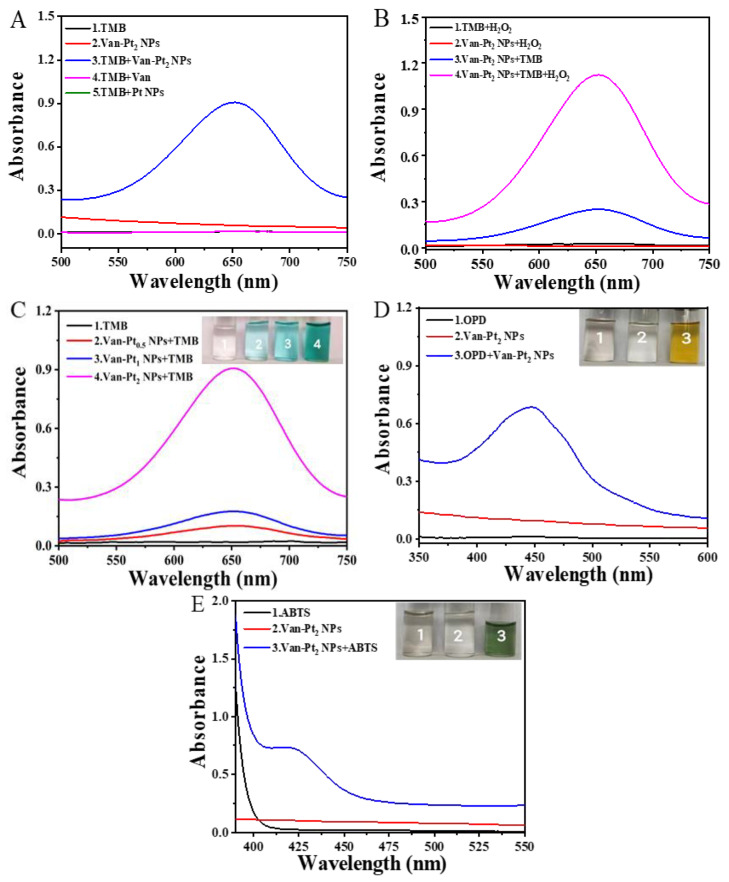
(**A**) Oxidase-like activity characterization, t = 5 min; (**B**) characterization of peroxidase-like activity, t = 2 min; (**C**) comparison of oxidase-like activity of Van-Pt_n_ NPs (the reaction time was 5 min); characterization of substrate (**D**) OPD and (**E**) ABTS.

To measure whether our synthesized Van-Pt_2_ NPs have oxidase-like activity, we designed five sets of experiments with 3,3′,5,5′-tetramethylbenzidine (TMB) for the oxidation substrate: (1) TMB; (2) Van-Pt_2_ NPs; (3) TMB + Van-Pt_2_ NPs; (4) TMB + Van; (5) TMB + Pt NPs. According to Figure 3A, group (3) TMB + Van-Pt_2_ NPs has the largest absorbance as 0.98 at 652 nm. The color of the samples in the group (3) of TMB + Van-Pt_2_ NPs changed from transparent to blue due to the oxidation of TMB to oxTMB. However, the absorbance of other groups was much lower than that of group (3). Moreover, Van-Pt_2_ NPs had higher absorbance for TMB reaction than bare Pt NPs and vancomycin, indicating that vancomycin modification enhanced the oxidase-like activity of Pt NPs. Thus, Van-Pt_2_ NPs have oxidase-like activity. It should be noted that the reaction time was 5 min. He et al. [26] prepared chondroitin sulfate-modified platinum nanozyme (CS-Pt NPs) exhibiting enhanced oxidase-like activity. The oxidase-like activity of CS-Pt NPs can be ascribed to the formation of the O_2_ center dot from the activation of dissolved O_2_.

To test whether Van-Pt_2_ NPs have peroxidase-like activity, we designed four groups of experiments: (1) TMB + H_2_O_2_; (2) Van-Pt_2_ NPs + H_2_O_2_; (3) TMB + Van-Pt_2_ NPs; (4) TMB + Van-Pt_2_ NPs + H_2_O_2_. It should be noted that the reaction time was 2 min. According to the experimental results in Figure 3B, the group (4) TMB + Van-Pt_2_ NPs + H_2_O_2_ has the largest absorbance as 1.18, which is much greater than that in group (3) TMB + Van-Pt_2_ NPs. In addition, the absorbance of group (1) TMB + H_2_O_2_ and group (2) Van-Pt_2_ NPs + H_2_O_2_ is close to 0. These results indicated that Van-Pt_2_ NPs had peroxidase-like activity. The difference of absorbance of TMB + Van-Pt_2_ NPs + H_2_O_2_ was due to different reaction times; H_2_O_2_ slowly breaks down into water and oxygen, which has a bad effect on its use. Thus, the oxidase-like activity of Van-Pt_2_ NPs without using H_2_O_2_ was carefully further studied. Van-Pt_n_ NPs with different molar ratios (n = 0.5, 1, and 2) reacted with TMB. It can be seen from Figure 3C that group (4) Van-Pt_2_ NPs + TMB has the highest absorbance, while the other groups demonstrate far lower absorbance value. Therefore, Van-Pt_2_ NPs exhibited the highest peroxidase-like activity.

To test whether Van-Pt_2_ NPs have the ability to oxidize different substrates, 1,2-diaminobenzene (OPD) and 2,2′-azinobis-(3-ethylbenzthiazoline-6-sulphonate) (ABTS) were used as substrates instead of TMB. The following experiments were designed: (1) OPD; (2) Van-Pt_2_ NPs; (3) Van-Pt_2_ NPs + OPD. ABTS was also used as a substrate to measure. As shown in Figure 3D,E, OPD was oxidized to orange-red oxidized OPD (oxOPD) with characteristic peaks at 448 nm in group (3) Van-Pt_2_ NPs + OPD, and ABTS was oxidized to blue oxidized ABTS (oxABTS) with characteristic peaks at 420 nm in group Van-Pt_2_ NPs + ABTS. Thus, the synthesized Van-Pt_2_ NPs have the ability to oxidize different substrates.

### 3.3. Optimal Catalytic Conditions and Catalytic Kinetics

**Figure 4:**
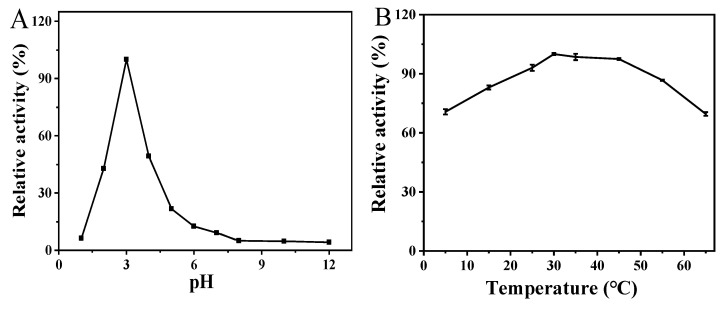

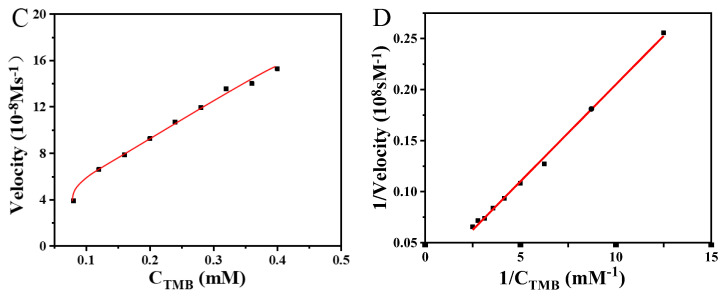
Optimal conditions for oxidase-like activity of Van-Pt_2_ NPs: (**A**) optimal pH; (**B**) optimal temperature; (**C**) catalytic kinetics of different TMB concentrations; (**D**) the double reciprocal plot of (**C**).

Similar to native enzymes and other nanomaterial-based oxidase-like nanozymes, the catalytic activity of the prepared Van-Pt_2_ NPs may be affected by external conditions such as pH and temperature. Therefore, we explored the optimal pH and temperature for oxidase-like activity of Van-Pt_2_ NPs. According to Figure 4A, the oxidase-like activity at pH = 3 was set to 100%. When pH = 2 and pH = 4, the oxidase-like activity of Van-Pt_2_ NPs decreased to 42.9% and 49.6%, respectively. Van-Pt_2_ NPs in other pHs showed very low oxidase-like activity. Thus, Van-Pt_2_ NPs had the highest oxidase-like activity at pH = 3. In Figure 4B, the activity of Van-Pt_2_ NPs reached its highest value at 30 °C. The activity of Van-Pt_2_ NPs decreased at other temperatures but remained at 69.6%. Thus, Van-Pt_2_ NPs have the highest oxidase-like activity when the temperature is 30 °C. Therefore, the optimum conditions for the enzymatic activity of Van-Pt_2_ NPs were pH = 3 and a temperature of 30 °C, and the subsequent experiments were performed under these conditions.

Therefore, the catalytic reaction kinetics of Van-Pt_2_ NPs were characterized to explore the oxidase-like activity. The reaction kinetics of the nanozyme was determined by varying the TMB concentration. The test data were then analyzed using the Lineweaver–Burk double reciprocal to obtain the data plot of Figure 4C,D. The Michaelis constant *K_m_* (mM) and the maximum reaction rate *V_max_* (Ms^−1^) were then calculated from the double reciprocal line. The standard equation for TMB was *y* = 0.01899x + 0.01512 (R^2^ = 0.9971), the substrate was TMB, the substrate concentration was 0.04–0.4 mM, and the *K_m_* and *V_max_* values of Van-Pt_2_ NPs were 1.256 mM and 66.138 × 10^−8^ Ms^−1^, respectively. It can be seen from Table 1 that Van-Pt_2_ NPs have obvious advantages over other nanozymes, such as Mn_0.6_Co_0.4_O MS [27], with a *V_max_* value of 7.62 × 10^−8^ Ms^−1^. This also indicates that Van-Pt_2_ NPs have excellent oxidase-like activity, which is beneficial for their catalytic reactions.

### 3.4. Stability of Van-Pt_2_ NPs

**Figure 5:**
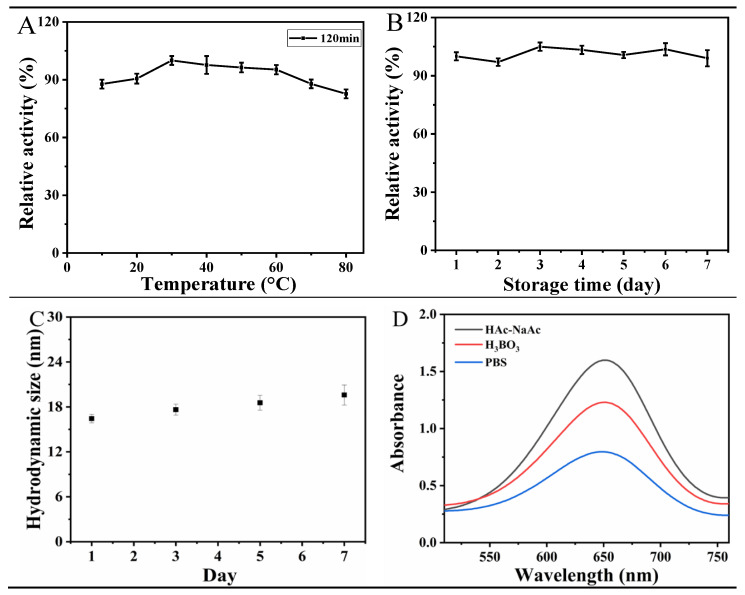
Stability determination of Van-Pt_2_ NPs: (**A**) temperature stability; (**B**) storage time stability; (**C**) hydrodynamic size variation with time; (**D**) the catalytic ability of Van-Pt_2_ NPs in different buffer solution.

After successful preparation of Van-Pt_2_ NPs nanozymes and verification of their biomimetic properties, the stability of Van-Pt_2_ NPs was tested, including temperature stability, storage stability, and salt solution stability. Figure 5A is the temperature stability of Van-Pt_2_ NPs. After incubation for 120 min in the range of 10 °C to 80 °C, Van-Pt_2_ NPs showed good catalytic performance in the range of 30 °C to 60 °C, while the catalytic performance increased with temperature in the range of 10 °C to 30 °C. After 60 °C, the catalytic performance gradually decreased to 78.6%. The oxidase-like activity of Van-Pt_2_ NPs maintained at a high level. Therefore, it can be concluded that Van-Pt_2_ NPs have good temperature stability.

Figure 5B shows the storage time stability test of Van-Pt_2_ NPs. With the increase in storage time, the oxidase-like activity of Van-Pt_2_ NPs remained around 100% with a fluctuation range of 97% to 105%. Therefore, the Van-Pt_2_ NPs have good storage stability. Figure 5C shows that the hydrodynamic size of Van-Pt_2_ NPs slowly grows by 3 nm with increasing storage time. Therefore, the synthesized Van-Pt_2_ NPs showed good storage stability. The catalytic properties of Van-Pt_2_ NPs were tested in different buffer solutions. The absorbance of Van-Pt_2_ NPs in HAc-NaAc buffer solution, H_3_BO_3_ buffer solution, and PBS buffer solution was 1.67, 1.25, and 0.79 in Figure 5D, respectively. The absorbance of Van-Pt_2_ NPs was highest in HAc-NaAc buffer solution. Therefore, Van-Pt_2_ NPs have the best oxidase-like activity in HAc-NaAc buffer solution.

### 3.5. Catalytic Mechanism of Van-Pt_2_ NPs

**Figure 6:**
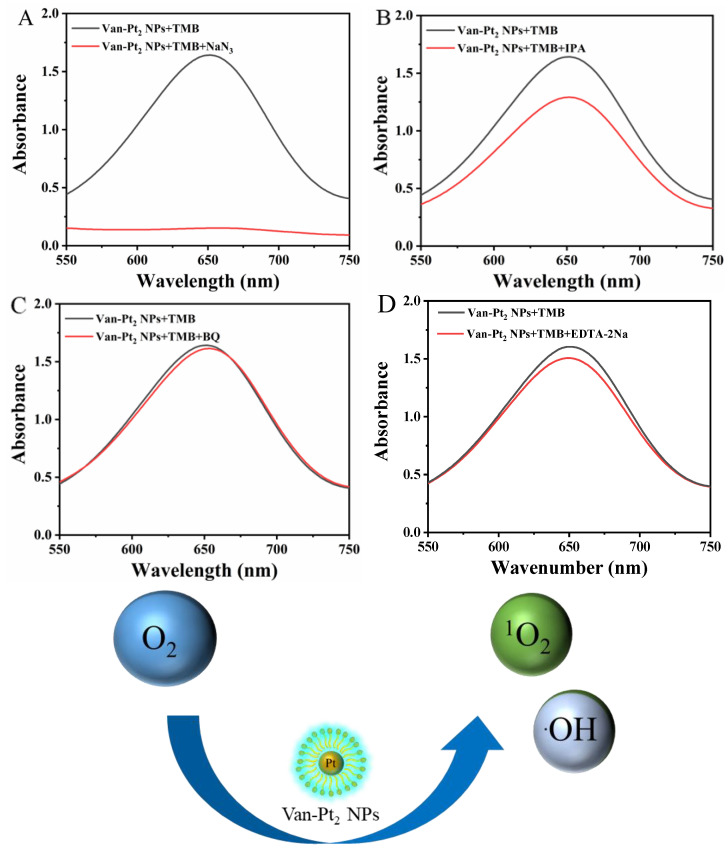
Oxidase-like activity mechanism of Van-Pt_2_ NPs: the absorbance spectra of Van-Pt_2_ NPs + TMB system with (**A**) NaN_3_, (**B**) IPA, (**C**) BQ, and (**D**) EDTA-2Na, respectively.

The oxidation of TMB is closely related to the species of reactive oxygen species (ROS). Therefore, it is necessary to explore the kinds of ROS produced in the oxidase-like reaction of Van-Pt_2_ NPs. Different ROS inhibitors—sodium azide (NaN_3_), isopropyl alcohol (IPA), ethylenediaminetetraacetic acid disodium salt (EDTA-2Na), and 1,4-Benzoquinone (BQ)—quench or inhibit ^1^O_2_, OH, h^+^, and O_2_^−^, respectively. In order to explore the types of ROS generated in the oxidase-like activity reaction of Van-Pt_2_ NPs, we set up five groups experiments: (1) Van-Pt_2_ NPs + TMB; (2) Van-Pt_2_ NPs + TMB + NaN_3_; (3) Van-Pt_2_ NPs + TMB + IPA; (4) Van-Pt_2_ NPs + TMB + EDTA-2Na; (5) Van-Pt_2_ NPs + TMB + BQ. Figure 6 shows that the absorbance of the reaction decreased by 90.7% after adding NaN_3_ to the Van-Pt_2_ NPs + TMB system. After adding IPA, the absorbance decreased by 21.3%. After the addition of BQ and EDTA-2Na, the absorbance decreased by 1.8% and 6.2%, respectively. These results indicated that ROS produced by the oxidase-like activity of Van-Pt_2_ NPs are mainly ^1^O_2_, containing a small amount of ·OH without h^+^ and O_2_^−^. Thus, the catalytic mechanism should be as follows: dissolved O_2_ was adsorbed and decomposed on the surface of Van-Pt_2_ NPs, and then the subsequently formed ^1^O_2_ and a small amount of OH by O_2_ can react with the substrate, thereby exerting the oxidase-like activity of Van-Pt_2_ NPs.

### 3.6. Detection of DA

**Figure 7:**
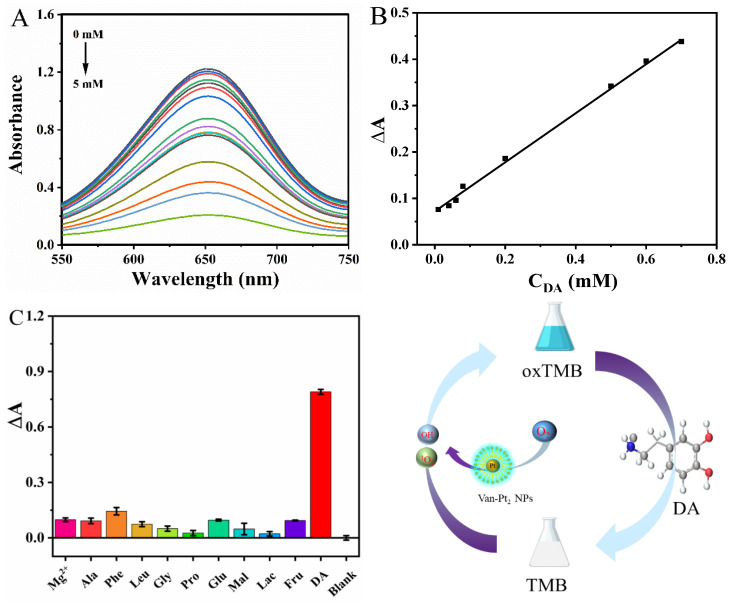
DA detection method by Van-Pt_2_ NPs: (**A**) UV–vis absorption spectra; (**B**) linear fit plots of Δ*A* over different concentrations; (**C**) detection of DA selectivity.

Van-Pt_2_ NPs have the ability to oxidize to TMB, and DA can reduce blue oxTMB to colorless TMB. Thus, we were able to construct a method to detect DA using the excellent oxidase-like activity of Van-Pt_2_ NPs. The experimental system is Van-Pt_2_ NPs + TMB + DA. As the concentration of dopamine (0–5 mM) gradually increased in the reaction system, the absorption intensity of the mixed solution at 652 nm gradually decreased (Figure 7A). At the same time, as shown in Figure 7B, Δ*A* (Δ*A* = *A*_blank_ − *A*_DA_) was linear with C_DA_ when the concentration of DA varied from 10 to 700 µM. The DA standard curve equation was established in the detection range of 10–700 µM: Y = 0.0713 + 0.53498 × C_DA_ (R^2^ = 0.9971), the slope and intercept were 0.53498 and 0.0713, respectively, and the limit of detection was 0.854 μM (LOD = 3σ/K). As shown in Table 2, compared with the reported SiW_9_Co_3_ [32] and h-CuS NCs [33] nanozymes, the detection range was 5.38–108 μM and 2–150 μM, respectively, and the detection limit was 5.38 μM and 1.67 μM, respectively. Therefore, Van-Pt_2_ NPs have a wider detection range and better sensitivity.

Other substances may affect the accuracy of DA detection, so it is necessary to test the anti-interference performance of Van-Pt_2_ NPs. The interfering agents included Mg^2+^,alanine (Ala), phenylalanine (Phe), leucine (Leu), glycine (Gly), proline (Pro), glutamic acid (Glu), maltose (Mal), lactose (Lac), and fructose (Fru). The concentration of DA was 3.33 mM, and the concentration of interfering agent was 10 mM. Figure 7C shows that the absorbance Δ*A* of the solution reached 0.80 when DA was added. When other interfering agents were added, the maximum absorbance Δ*A* of the solution was only 0.14. Therefore, Van-Pt_2_ NPs had anti-interference performance for DA detection.

Based on the accuracy and selectivity of Van-Pt_2_ NPs, the recovery rate of DA in real samples was studied. The recovery rate of the sample was calculated using the DA standard curve equation, and the recovery rate of DA was 99–110% (Table 3).

## 4. Conclusions

In summary, we successfully synthesized a kind of nanozyme with high activity of oxidase-like and peroxidase-like activity via biological template method. The synthesized Van-Pt_2_ NPs had good thermal stability and storage time stability. The catalytic kinetics of Van-Pt_2_ NPs conformed to the typical Michaelis–Menten equation. Moreover, a simple, fast, and reliable method for DA detection was established based on the oxidase-like activity of Van-Pt_2_ NPs. The detection range was 10–700 µM, and the detection limit was 0.854 μM. In addition, the MTT assay showed that Van-Pt_2_ NPs and vancomycin almost had no cytotoxicity to A549 cells. Therefore, we synthesized Van-Pt_2_ NPs with multiple functionalities, good stability, and good biocompatibility.

## Figures and Tables

**Table 1:** Comparison of the kinetic parameters *K_m_* and *V_max_*.

Materials	Substrate	*K*_m_ (mM)	*V*_max_ (×10^−8^ Ms^−1^)	Reference
Van-Pt_2_ NPs	TMB	1.256	66.138	this work
Mn_0.6_Co_0.4_O MS	TMB	0.0187	7.62	[27]
Se NPs	TMB	8.3	5.07	[28]
Ru@V_2_O_4_ NWs	TMB	0.045	10.9	[29]
NiPd hNPs	TMB	0.11	1.52	[30]
HRP	TMB	0.43	10	[31]

**Table 2:** Comparison of DA detection range and detection limit of different materials.

Materials	Detection Method	Linear Range (μM)	LOD (μM)	Reference
Van-Pt_2_ NPs	Colorimetry	10–700	0.854	this work
SiW_9_Co_3_	Colorimetric	5.38–108	5.38	[32]
h-CuS NCs	Colorimetric	2–150	1.67	[33]
Pt/hBN NSs	Colorimetric	2–50	0.76	[34]
CDS	Fluorescence	3.0–20	1.0	[35]

**Table 3:** Detects the recovery of DA in tap water and sea water.

Sample	Added DA Concentration (μM)	Found DA Concentration (μM)	Recovery (%)	RSD (%)
Tap water Seawater	500	495	99	0.254
	500	550	110	1.95

## Data Availability

The data presented in this study are available on request from the corresponding author.

## Supplementary Materials

The following supporting information can be downloaded at https://www.mdpi.com/article/10.3390/biom13091312/s1: Electronic Supporting Information (ESI) is available: Preparation of Van-Pt_n_ NPs and Pt NPs; Enzyme-like activity assay; Optimal conditions; Catalytic kinetics; Stability test; Activity mechanism; Dopamine detection; Figure S1: XPS and XRD characterization; Figure S2: Biocompatibility test. References [36,37] are cited in the supplementary materials.
