# Supplementary material for: Vancomycin-Stabilized Platinum Nanoparticles with Oxidase-like Activity for Sensitive Dopamine Detection
Source: Biomolecules. 2023 Aug 26;13(9):1312. doi: 10.3390/biom13091312 (PMC10527023; doi:10.3390/biom13091312)
Supplement: Supplementary file 1 [file biomolecules-13-01312-s001.zip › biomolecules-2512036-supplementary.pdf]

Supplementary Information

Experimental section

Materials and instruments

Van·HCl, potassium tetrachloroplatinate(II) (K_2PtCl_4), hydrogen peroxide (H_2O_2), 3,3',5,5'-tetramethylbenzidine (TMB), sodium borohydride ($NaBH_4$), 3-hydroxytyramine hydrochloride (DA·HCl), thiazole blue (MTT), 1,2-diaminobenzene (OPD), sodium azide (NaN_3), isopropyl alcohol (IPA) were purchased from Aladdin Reagent Co., Ltd. Sodium tetrachloropalladate(II) (Na_2PdCl_4) was purchased from Josiah reagent Co., Ltd. 2,2'-azinobis-(3-ethylbenzthiazoline-6-sulphonate) (ABTS) was purchased from Nanjing Jiru Biotechnology Co., Ltd. A549 cells were purchased from Center for Typical Culture Collection.

Absorbance was measured using a TU1810 spectrometer. The morphology and size of the nanoparticles were observed by transmission electron microscope (HT 7700). The elemental composition and valence states of the nanoparticles were analyzed by X-ray photoelectron spectroscopy (Kratos AXIS Ultra). X-ray diffraction (XRD) peaks were obtained with an X-ray diffractometer (D-max-2500/PC). The zeta potential and hydrodynamic particle size of the nanoparticles were obtained using a laser particle size meter (Zetasizer Nano-ZS90). The biocompatibility was tested with a multifunction microplate reader (SpectraMax M2).

Preparation of Van-Pt_n NPs

Van-Pt_n NPs were prepared according to the following procedure. 73 μL Van·HCl (10 mM) solution was added to the centrifuge tube, followed by various amounts of

K_2PtCl_4 (10 mM) solution, the ratio of vancomycin to K_2PtCl_4 is 1:0.5, 1:1, 1:2, and 1:3, respectively. Then, 1000 μL deionized water was added. The mixed solution was incubated for 12 h at 25°C. 10 μL $NaBH_4$ solution (1 M, dissolved in 0.3 M NaOH solution) was added, and then the mixed solution was placed in a constant temperature mixer for 12 h., Van-Pt_n NPs were obtained after dialysis.

For the preparation of Pt nanoparticles (Pt NPs), 146 μL K_2PtCl_4 (10 mM) solution was added to a 2 mL centrifuge tube, and then 1073 μL deionized water was added, the mixed solution was incubated for 12 h at 25°C. Afterwards, 10 μL $NaBH_4$ solution (1 M, dissolved in 0.3 M NaOH solution) was added and incubated for 12 h. Pt NPs were obtained after dialysis.

Enzyme-like activity assay

Oxidase-like activity was determined according to the following procedure. 200 μL Van-Pt_n NPs (n= 0.5, 1, 2) (C_{Pt}=0.9 mM) was added to a 2 mL centrifuge tube. 300 μL NaAc-HAc buffer solution (0.2 M) and 1000 μL NaAc-HAc buffer solution (0.2 M) containing 0.6 mM TMB were added. The mixed solution was incubated at 25°C for 5 min. Finally, the absorbance was determined by UV-vis spectrometer.

Peroxidase-like activity was also measured. 200 μL Van-Pt_n NPs (n= 0.5, 1, 2) (C_{Pt}=0.9 mM) was added to a 2 mL centrifuge tube. Added 300 μL NaAc-HAc buffer (0.2 M,) and 1000 μL NaAc-HAc buffer (0.2 M, including 0.6 mM TMB). Then 100 μL H_2O_2 solution (0.03 M) was added. The mixed solution was incubated at 25°C for 5 min. Finally, the absorbance was determined by UV-vis spectrometer.

The chromogenic substrate was changed to OPD (50 mM) and ABTS (50 mM),

the mixed solution was incubated at 25°C for 5 min. Finally, the absorbance was determined by UV-vis spectrometer.

Optimal conditions

To find the best pH, 200 μL Van-Pt_2 NPs (C_{Pt}=0.9 mM) was added to a 2 mL centrifuge tube, and then 300 μL of NaAc-HAc buffer solution with different pH (1-12) was added. Then 1000 μL of NaAc-HAc buffer solution (0.2 M) containing 0.6 mM TMB was added. The samples were incubated for 5 min and the absorbance at 652 nm was measured by UV-vis spectrometer. Three control groups were set at the same time.

In addition, the optimal temperature of the nanozymes was explored by setting different temperature conditions, 200 μL Van-Pt_2 NPs (C_{Pt}=0.9 mM) was added to a 2 mL centrifuge tube, and then 300 μL NaAc-HAc buffer solution (0.2 M) was added. Finally, 1000 μL of NaAc-HAc buffer solution (0.2 M) containing 0.6 mM TMB was added. The prepared experimental solution was incubated in a constant temperature mixer at different temperatures (5-65°C) for 5 min. Finally, the absorbance was measured using UV-vis spectrometer. Three control groups were set at the same time.

Catalytic kinetics

200 μL Van-Pt_2 NPs (C_{Pt}=0.9 mM) was added to a 2 mL centrifuge tube. 300 μL NaAc-HAc buffer solution (0.2 M) and 1000 μL NaAc-HAc buffer solution (0.2 M) containing 0.6 mM TMB were added. The mixed solution was incubated at 25°C for 5 min. The time- dependent absorbance at 652 nm was then tested using a UV-vis spectrometer. The amount of buffer solution was 1200-300 μL. The amount of buffer solution containing TMB was 100 to 1000 μL. The total sollution in the centrifuge tube

was 1500 μL. The Michaelis-Menten constant (K_m)and maximum reaction rate (V_{max}) were studied by the kinetic **Formula S1**[1]:

$$v = \frac{V_{\mathrm{max}}[\mathrm{S}]}{K_{\mathrm{m}}+[\mathrm{S}]} \qquad \text{(S1)}$$

where K_m means the Michaelis constant, V_{max} means the maximum reaction rate, and [S] means the substrate concentration.

Stability test

To determine the temperature stability, 200 μL Van-Pt_2 NPs (C_{Pt}=0.9 mM) was added to a 2 mL centrifuge tube. Then, they were placed in a constant temperature mixer (600 r/min) at different temperatures for 2 h, and the temperature ranged from 10-90°C. Following that 300 μL NaAc-HAc buffer solution (0.2 M, pH=4) was added. Finally, 1000 μL of NaAc-HAc buffer solution (0.2 M, pH=4) containing 0.6 mM TMB was added. The prepared solution was placed in a constant temperature mixer at 25°C and 600 r/min for 5 min. Finally, the absorbance was measured using UV-vis spectrometer. Three control groups were set at the same time.

The storage stability was also measured, Van-Pt_2 NPs were stored in the refrigerator at 4°C. 200 μL Van-Pt_2 NPs (C_{Pt}=0.9 mM) was added to a 2 mL centrifuge tube. Subsequently, 300 μL of NaAc-HAc buffer solution (0.2 M, pH=4) and 1000 μL of NaAc-HAc buffer solution (0.2 M, pH=4) containing 0.6 mM TMB were sequentially introduced. The mixed solution was incubated at 25°C for 5 min. Finally, the absorbance was determined by UV-vis spectrometer. Three control groups were set at the same time.

The stability of the nanozyme was also compared in different buffer solutions,

including NaAc-HAc buffer solution, phosphate buffer solution and borate buffer solution. 200 μL Van-Pt_2 NPs (C_{Pt}=0.9 mM) was added to a 2 mL centrifuge tube. Afterwards, 300 μL buffer solution (0.2 M, pH=4) was introduced, followed by the addition of 1000 μL of buffer solution (0.2 M, pH=4) containing 0.6 mM TMB. The samples were incubated for 5 min and then the absorbance was determined. Three control groups were set at the same time.

Activity mechanism

Exploration of the mechanism of oxidase-like activity: 200 μL Van-Pt_2 NPs (C_{Pt}=0.9 mM) was added to a 2 mL centrifuge tube, followed by 1000 μL NaAc-HAc buffer solution (0.2 M, pH= 3) containing 0.6 mM TMB. Then 200 μL solutions of different reactive oxygen species (ROS) inhibitors were added. The experimental samples were incubated in the constant temperature mixer at 30ºC and 600 r/min for 5 min. Finally, UV-vis spectrometer was used to determine the absorbance of the sample at 500 to 750 nm. ROS inhibitors were p-benzoquinone (BQ), sodium azide (NaN_3), isopropyl alcohol (IPA) and disodium ethylenediamine tetraacetate (EDTA-2Na), and their concentration was 10 mM.

Dopamine detection

50 μL Van-Pt_2 NPs (C_{Pt}=0.9 mM) was added to a 2 mL centrifuge tube, followed by 1000 μL NaAc-HAc buffer solution (0.2 M, pH= 3) containing 0.6mM TMB. Then added 200 μL different concentrations of DA (0-5 mM) solution. The mixed solution was incubated at 25ºC and 600 r/min for 5 min. Finally, the absorbance was measured by UV-vis spectrometer. The relationship between absorbance difference and

concentration is used to make the standard curve of detection. Three control groups were set at the same time.

50 μL Van-Pt_2 NPs (C_{Pt}=0.9 mM) was added to a 2 mL centrifuge tube, followed by 1000 μL NaAc-HAc buffer solution (0.2 M, pH= 3) containing 0.6 mM TMB. Then 200 μL of different scalars of solution was added. The configured solution was mixed and incubated for 5 min in a constant temperature mixer at 25°C and 600 r/min. Finally, the absorbance was measured by UV-vis spectrometer and substituted into the standard curve. The spiked recovery **Formula S2** [2] was used to calculate the recovery of the sample.

$$P = \frac{(Y_0\text{-}Y_1)\text{-}a}{b\times c} \times 100\% \qquad \text{(S2)}$$

where P means the Spiked recovery rate, Y_0 means unspiked absorbance, Y_1 means spiked absorbance, a is the intercept of the standard curve, b is the slope of the standard curve, and c is the scalar. Three control groups were set at the same time.

Different interferences were added to the test tubes, and the anti-interference ability of the nanozymes was judged by the absorbance. The specific experimental procedure was as follows: 50 μL Van-Pt_2 NPs (C_{Pt}=0.9 mM) was added to a 2 mL centrifuge tube, followed by 1000 μL NaAc-HAc buffer solution (0.2 M, pH= 3) containing 0.6 mM TMB. Then 200 μL of aqueous solution containing different interfering agents was added. Three control groups were set at the same time.

Biocompatibility test

The biocompatibility of Van-Pt_2 NPs was determined by MTT assay. Firstly, the cells were added to the 96-well plate and incubated in the cell incubator for 24 h. Then,

the original medium was replaced with high glucose medium containing Van-Pt_2 NPs, and incubated for 24 h. Following this, the medium was replaced with thiazolyl blue solution and incubated for 4 h. Finally, dimethyl sulfoxide (DMSO) was added into the solution, and the absorbance of the 96-well plate was measured by microplate reader. Three control groups were set at the same time.

XPS and XRD characterization

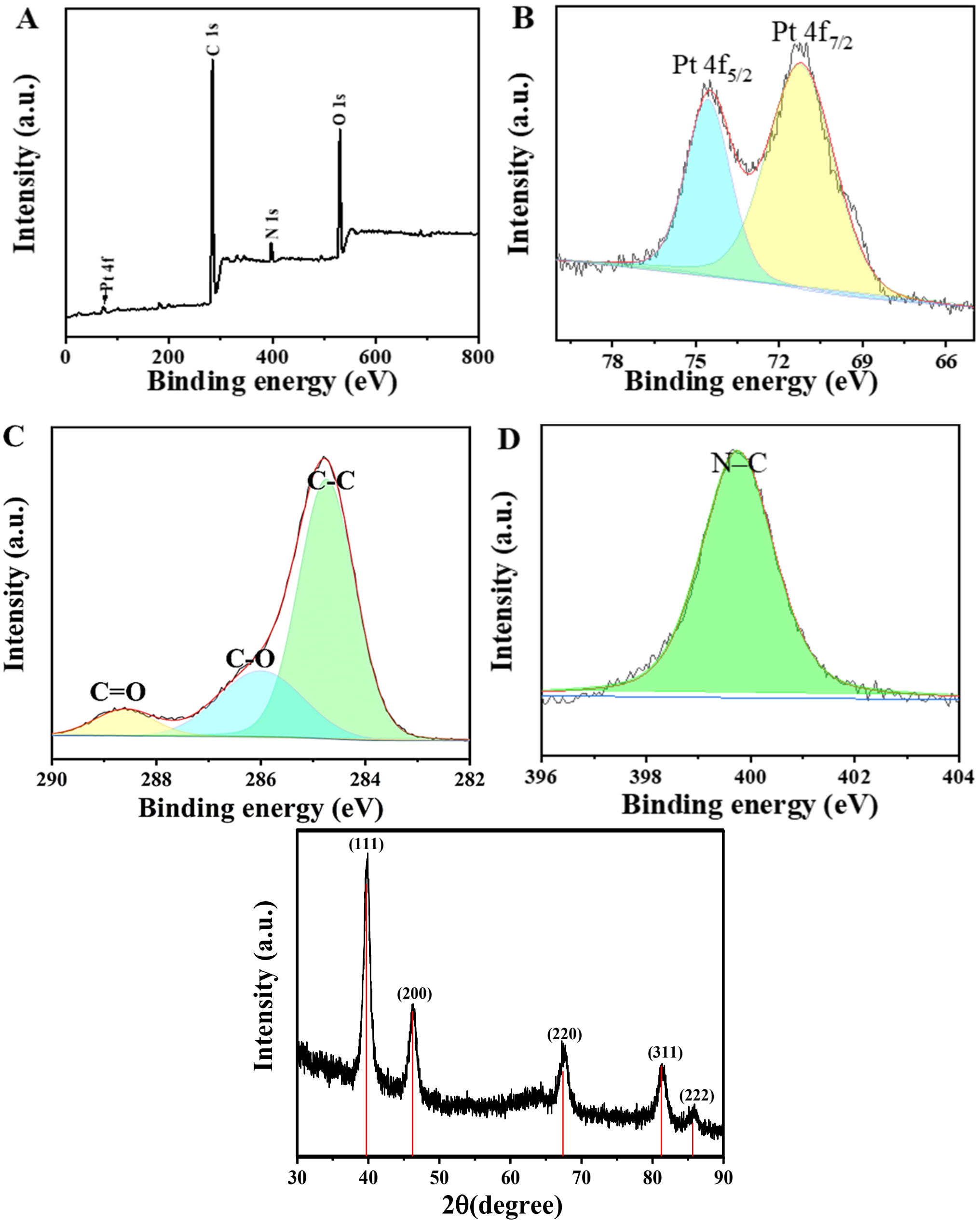

Figure S1. (A) Full XPS spectrum of Van-Pt_2 NPs; high resolution XPS spectra of Van-Pt_2 NPs: (B) Pt 4f; (C)C 1s; (D)N 1s ； (E) XRD image of Van-Pt_2 NPs

Biocompatibility analysis

Van-Pt_2 NPs exhibited similar catalytic activity to native enzymes. It is necessary not only to test the enzyme-like activity of Van-Pt_2 NPs, but also to determine the biocompatibility of Van-Pt_2 NPs, so the cytotoxicity of Van-Pt_2 NPs was evaluated by MTT assay. The biocompatibility of the Van-Pt_2 NPs was determined by measuring the cell viability of the Van-Pt_2 NPs after incubation for 24 h. As shown in **Figure S2**, Van-Pt_2 NPs group and vancomycin group were almost non-cytotoxic, and the cell viability remained above 90% within the concentration range. Since vancomycin is zwitterionic and has good biocompatibility, the biocompatibility of Van-Pt_2 NPs has been improved. Vancomycin contains nine hydroxyl groups, two amino groups and one carboxyl group in a charge balancing condition.

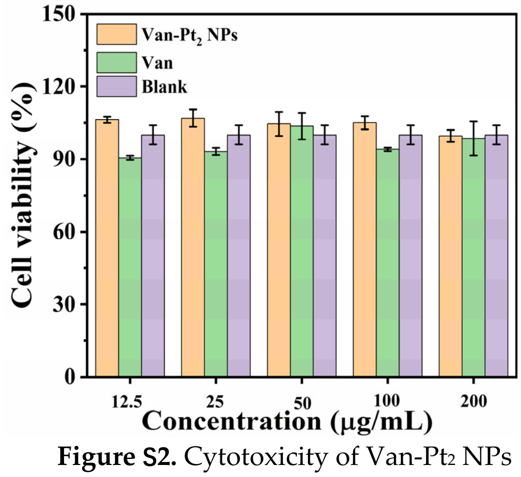

Figure S2. Cytotoxicity of Van-Pt_2 NPs

References

1. Dong, J. S. On catalytic kinetics of enzymes. *Processes*. **2021**, *9*, 271.
2. Chen, J.; Lu, Y.; Yan, F.; Wu, Y.; Huang, D.; Weng, Z., A fluorescent biosensor based on catalytic activity of platinum nanoparticles for freshness evaluation of aquatic products. ***Food Chemistry***. **2020**, *310*, 125922.
